# Bioinformatics analysis of aging-related genes in thoracic aortic aneurysm and dissection

**DOI:** 10.3389/fcvm.2023.1089312

**Published:** 2023-05-22

**Authors:** Hong Wan, Danlingyi Liu, Bingqing Liu, Mengyao Sha, Wei Xia, Chang Liu

**Affiliations:** School of Medical Technology, Beihua University, Jilin, China

**Keywords:** aging, thoracic aortic aneurysm and dissection, bioinformatics analysis, single-cell RNA sequencing, biomarkers

## Abstract

**Objective:**

Thoracic aortic aneurysm and dissection (TAAD) is a cardiovascular disease with a high mortality rate. Aging is an important risk factor for TAAD. This study explored the relationship between aging and TAAD and investigated the underlying mechanisms, which may contribute to the diagnosis and treatment of TAAD.

**Methods:**

Human aging genes were obtained from the Aging Atlas official website. Various datasets were downloaded from the GEO database:the human TAAD dataset GSE52093 were used for screening differentially expressed genes (DEGs); GSE137869, GSE102397 and GSE153434 were used as validation sets, and GSE9106 was used for diagnostic prediction of receiver operating characteristic (ROC) curves. Gene Ontology (GO), Kyoto Encyclopedia of Genes and Genomes (KEGG), Gene Set Enrichment Analysis (GSEA), and protein–protein interaction (PPI) network analysis were used to screen differentially co-expressed genes from human aging genes and TAAD. Using five methods of the cytoHubba plugin in Cytoscape software (Degree, Closeness, EPC, MNC, Radiality), hub genes were identified from the differentially co-expressed genes. Single-cell RNA sequencing was used to verify the expression levels of hubgenes in different cell types of aortic tissue. ROC curves were used to further screen for diagnostic genes.

**Results:**

A total of 70 differentially co-expressed genes were screened from human aging genes and DEGs in human TAAD dataset GSE52093. GO enrichment analysis revealed that the DEGs played a major role in regulating DNA metabolism and damaged DNA binding. KEGG enrichment analysis revealed enrichment in the longevity regulating pathway, cellular senescence, and HIF-1 signaling pathway. GSEA indicated that the DEGs were concentrated in the cell cycle and aging-related p53 signaling pathway. The five identified hubgenes were *MYC*, *IL6*, *HIF1A*, *ESR1*, and *PTGS2*. Single-cell sequencing of the aging rat aorta showed that hubgenes were expressed differently in different types of cells in aortic tissue. Among these five hubgenes, *HIF1A* and *PTGS2* were validated in the aging dataset GSE102397; *MYC*, *HIF1A* and *ESR1* were validated in the TAAD dataset GSE153434. The combined area under the diagnostic ROC curve (AUC) values for the five hub genes were >0.7 in the testing and training sets of the dataset GSE9106. The combined AUC values of *MYC* and *ESR1* were equal to the combin ed AUC values of the five hub genes.

**Conclusion:**

The HIF-1 signaling pathway may play an important role in TAAD and aging. *MYC* and *ESR1* may have diagnostic value for aging-related TAAD.

## 1. Introduction

Thoracic aortic aneurysms and dissection (TAAD) is a serious cardiovascular disease (CVD) with early presentation of symptoms, rapid onset and development, and a high incidence rate and mortality rate (1). Understanding the pathogenesis of TAAD is crucial for the diagnosis and treatment of TAAD. TAAD predominantly occurs in people older than 60 years, thus aging is a major risk factors for TAAD (2, 3). A growing body of research shows that aging plays a vital role in TAAD (4, 5).

Aging is a biological phenomenon in which the structure and function of an organism decline over time. Senescence has recently been discovered to be instrumental in aging and has been implicated as a major cause of age-related disease. In mammals, aging is associated with the accumulation of senescent cells. During the aging process, cells experience mitochondrial damage, DNA damage, cellular stress [reactive oxygen species (ROS)] upregulation of p53/p21 and p16 signaling (6–8), and various other mechanisms that contribute to the development and progression of CVD (9). In addition, vascular aging is associated with senescence, telomere shortening, and epigenetic changes in vascular endothelial (EC) and smooth muscle cell (SMC), which affect cell function and morphology. Aging and telomere shortening are also characteristic of aneurysmal tissue (10). The presence of numerous aging SMCs leads to excessive excessive inflammation and deformation of the aorta, promoting TAAD formation (4). Tyrrell et al. (11) reported that differentially expressed proteins in aged versus young aortic tissue were also differentially expressed in the aortic tissue of patients with TAA compared with healthy controls, proteomics revealed that aging transformed the aorta quantitatively and qualitatively from a healthy status to TAA. The relationship between aging and TAAD and its mechanism of action require further study. Exploring aging-related genes in TAAD will provide potential therapeutic targets and biomarkers for TAAD treatment.

In this study, the differential expression of aging-related genes in TAAD were assessed using the Gene Expression Omnibus (GEO) database. Differentially expressed genes (DEGs) underwent enrichment analysis with Gene Ontology (GO), Kyoto Encyclopedia of Genes and Genomes (KEGG), and Gene Set Enrichment Analysis (GSEA). Protein-protein interaction (PPI) and correlation analysis were then performed to explore the mechanism of aging in TAAD. Based on the single-cell sequencing results of the aged rat aorta, the expression level of hubgenes in different aortic tissue cells was investigated. In addition, receiver operating characteristic (ROC) curve expression levels of key DEGs were validated to provide a basis for gene prediction.

## 2. Materials and methods

### 2.1. Genetic data sources

Various datasets were collected from the GEO database (https://www.ncbi.nih.gov/geo/). GSE52093 includes ascending aorta gene expression data in patients with acute Stanford type A aortic dissection (*n* = 7) and healthy controls (*n* = 5); GSE153434 includes ascending aorta gene expression data in patients with Stanford type A aortic dissection (*n* = 10) and healthy controls (*n* = 10); GSE102397 includes vascular mRNA expression data from 3 to 4-month-old mice (*n* = 7) and 12-month-old mice (*n* = 6); GSE71226 includes patients with coronary heart disease (*n* = 3) and healthy people (*n* = 3); GSE9128 includes three patient groups:control subjects (*n* = 12) were divided into C1, C2, and C3; ischemic cardiomyopathy (ICM) patients (*n* = 12) were divided into ICM1, 2, 3, and 4; and nonischemic dilated cardiomyopathy (NIDCM) patients (*n* = 12) were divided into NIDCM1, 2, 3, and 4; and GSE12480 includes old mice (*n* = 10) and young mice (*n* = 10). GSE137869 is the single-cell RNA sequencing data of nine different rat tissue cells, and includes young (age 5 months) rats (*n* = 6, 3 male and 3 female rates) and old (age 27 months) rats (*n* = 6, 2 male and 4 female rates); the single-cell RNA sequencing data of the rat aorta, including the young group and the old group, were selected from this dataset. GSE9106 is a genome-wide gene expression profile of peripheral blood samples (collected from 59 patients with confirmed TAA and 34 healthy controls), including a training set (36 patients with TAA and 25 healthy controls) and a test set (23 patients with TAA and 9 healthy controls). Three of the datasets were missing grouping information and were removed from the subsequent analysis. Information on the mentioned datasets were shown in Table 1. Human aging genes were compiled from the official website of the Aging Atlas (12) (https://ngdc.cncb.ac.cn/aging/index).

**Table 1:** Dataset information table.

Dataset	Organism	Experimental cohort	Control cohort	Use(s) in this study
GSE52093	*Homo sapiens*	Aorta dissected patients (*n* = 7)	Healthy controls (*n* = 5)	Screening of DEGs, PCA analysis, Functional enrichment analysis, PPI analysis
GSE137869	*Rattus norvegicus*	27-month-old rats (*n* = 6)	5-month-old rats (*n* = 6)	Single-cell RNA sequencing
GSE102397	*Mus musculus*	12-month-old mice (*n* = 6)	3–4-month-old mice (*n* = 7)	Gene expression verification in aging
GSE153434	*Homo sapiens*	Stanford type A aortic dissection patients (*n* = 10)	Healthy controls (*n* = 10)	Gene expression verification in TAAD
GSE71226	*Homo sapiens*	Patients with coronary heart disease (*n* = 3)	Healthy people (*n* = 3)	Gene expression verification in coronary heart disease
GSE9128	*Homo sapiens*	Ischemic cardiomyopathy (ICM) (*n* = 12); non-ischemic cardiomyopathy (NICM) (*n* = 12)	Controls (*n* = 12)	Gene expression verification in heart failure
GSE12480	*Mus musculus*	25–28-month-old mice (*n* = 10)	4–6-month-old mice (*n* = 10)	Gene expression verification in aging cardiac tissue
GSE9106	*Homo sapiens*	TAA patients (*n* = 59)	Healthy controls (*n* = 34)	ROC curve analysis

### 2.2. Screening of DEGs

GSE52093 was screened for DEGs using the data analysis tool GEO2R (https://www.ncbi.nlm.nih.gov/geo/geo2r/) in the GEO database. According to *P* < 0.05, |log_2_FC| > 1, DEGs were screened, and 1,558 genes were obtained, including 794 up-regulated genes and 764 down-regulated genes. A total of 500 human aging-related genes were downloaded from the Aging Atlas (https://ngdc.cncb.ac.cn/aging/index). Finally, 70 DEGs related to aging were obtained from the intersection.

### 2.3. Differential expression analysis of aging-related genes

The gene expression differences between patients with acute Stanford type A aortic dissection and healthy controls in the GSE52093 dataset were analyzed with principal component analysis (PCA) in R software, and the results were visualized as a volcano plot, heatmap, and boxplots drawn with the “ggplot2” package.

### 2.4. Functional enrichment analysis of aging-related DEGs

GO and KEGG enrichment analysis of DEGs was performed using the “clusterProfiler” package in R software (13). GO analysis consisted of biological process (BP), cellular component (CC), and molecular function (MF). The up and downregulated genes of GSE52093 were subjected to GSEA and the results were visualized using the “ggplot2” package.

### 2.5. PPI and correlation analysis of aging-related DEGs

PPI analysis of aging-related DEGs was performed using the String (https://string-db.org/cgi/) database and Cytoscape software (version 3.8.2). Correlation analysis of aging-related DEGs was performed using Spearman correlation in the “ggplot2” package.

### 2.6. Hubgenes and their interactions

Five ranking methods of the cytoHubba plugin (Degree, Closeness, EPC, MNC, Radiality) in Cytoscape software were used to rank DEGs, then the top 10 DEGs were screened for overlapping genes and the top five overlapping genes were considered as hubgenes. NetworkAnalyst3.0 (https://www.networkanalyst.ca/) is a comprehensive network visualization analysis platform for gene expression analysis (14). This platform was used to analyze interactions between hubgenes. Hub gene–disease association information was shown using the DisGeNet database (version 7.0) (https://www.disgenet.org), which is only applicable to human data. Hub gene–drug interactions were shown using the DrugBank database (version 5.0) (http://go.drugbank.com), which is also only applicable to human data.

### 2.7. Distribution and expression of hubgenes in the aorta of aging rats verified by single-cell RNA sequencing

Using the GSE137869 single-cell sequencing dataset, four aortic samples—F-O (*n* = 4), F-Y (*n* = 3), M-O (*n* = 2), and M-Y (*n* = 3) (female old mice, female young mice, male old mice, and male young mice respectively)—were selected for single-cell sequencing analysis. Data processing and methods for clustering and identification of cell types are detailed in the original literature (15).

### 2.8. Validation of hubgenes in aging and TAAD

Gene expression was verified in the aging-related datasets GSE102397 and the TAAD-related dataset GSE153434. In addition, gene expression was verified in the coronary heart disease-related dataset GSE71226, the heart failure-related dataset GSE9128, and the aging cardiac tissue dataset GSE12480. Gene expression maps were drawn with the “ggplot2” package.

### 2.9. Diagnostic prediction of hubgenes

Using the dataset GSE9106 (testing set and training set), the ROC curve analysis and result visualization of the hubgenes were performed with the “pROC” package and “ggplot2”, respectively.

### 2.10. Statistical analysis

Differences between normally distributed variables were assessed using the *t*-test and differences between non-normally distributed variables were assessed using the Wilcoxon rank sum test. For all statistical tests, *P *< 0.05 was statistically significant.

## 3. Results

### 3.1. Differential expression analysis of aging-related genes in TAAD

**Figure 1:**
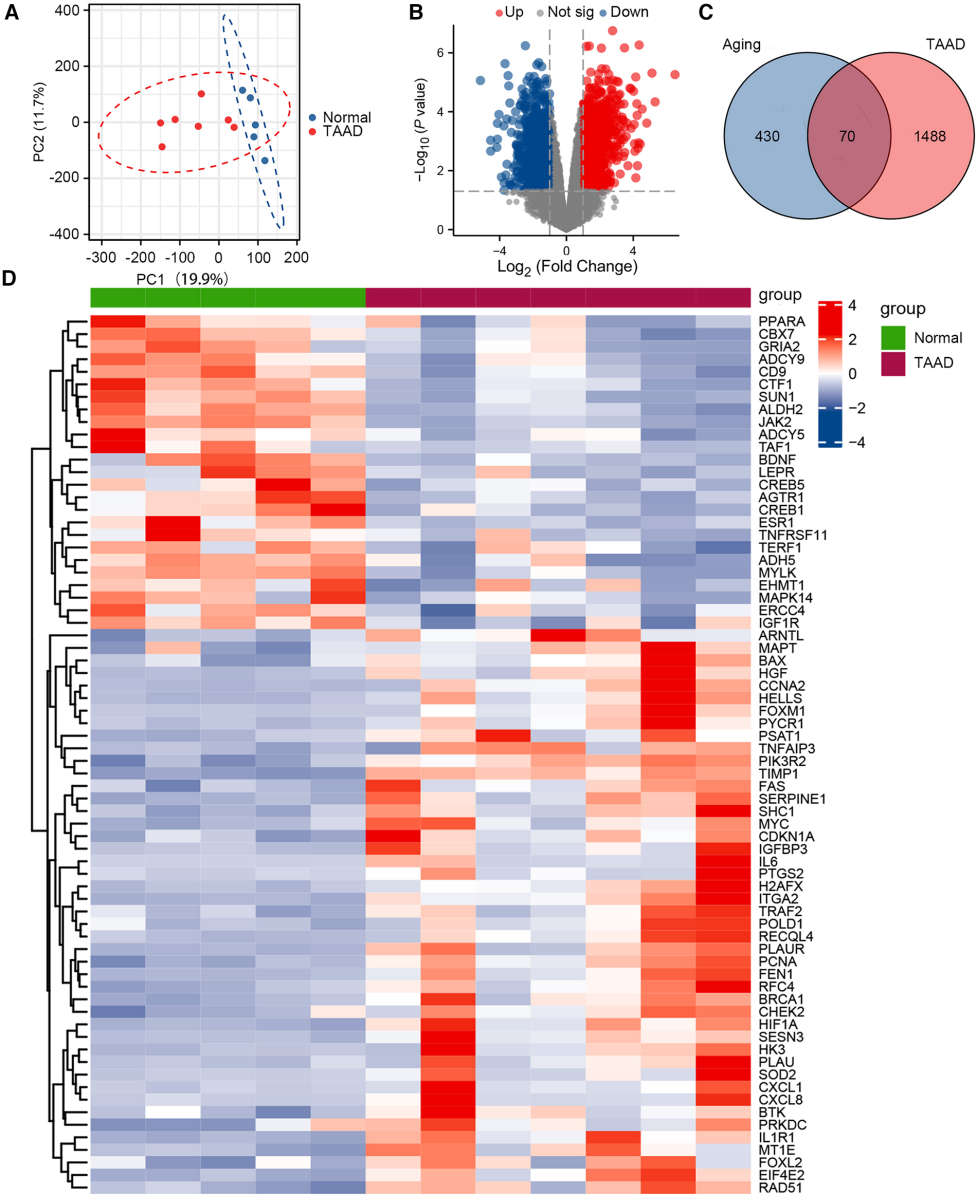
Differential expression of aging-related genes in TAAD. (**A**) Principal component analysis of GSE52093. (**B**) Volcano plot of differentially expressed genes (DEGs) in GSE52093. Red dots represent significantly upregulated genes and blue dots represent significantly downregulated genes. (**C**) Venn diagram of DEGs between human aging-related genes and TAAD. (**D**) Heatmap of 70 aging-related DEGs in TAAD.

The GSE52093 dataset is a genome-wide analysis of gene expression in the ascending aorta of patients with acute Stanford type A aortic dissection (*n* = 7) and normal controls (*n* = 5). To assess the reproducibility of the data within groups, PCA was performed. The data in GSE52093 was confirmed to have good reproducibility; PC1 of the GSE52093 dataset reflects 19.9% and PC2 reflects 11.7% of the variation in data characteristics (Figure 1A). Next, GEO2R was used to analyze the GSE52093 dataset according to *P* < 0.05, |log_2_FC| > 1, which identified 1,558 genes, including 794 upregulated genes and 764 downregulated genes. A volcano map was drawn using the “ggplot2” package in R software (Figure 1B). A total of 500 human aging-related genes were downloaded from the Aging Atlas and took the intersection with TAAD-related genes was taken to obtain 70 aging-related DEGs, including 45 upregulated genes and 25 downregulated genes. A Venn diagram was produced using the “ggplot2” package (Figure 1C). A heatmap of the 70 aging-related DEGs in TAAD was generated using the “ggplot2” package (Figure 1D). In addition, boxplots were produced showing the expression of the 70 aging-related up- and downregulated DEGs in TAAD (Supplementary Figure S1).

**Figure 2:**
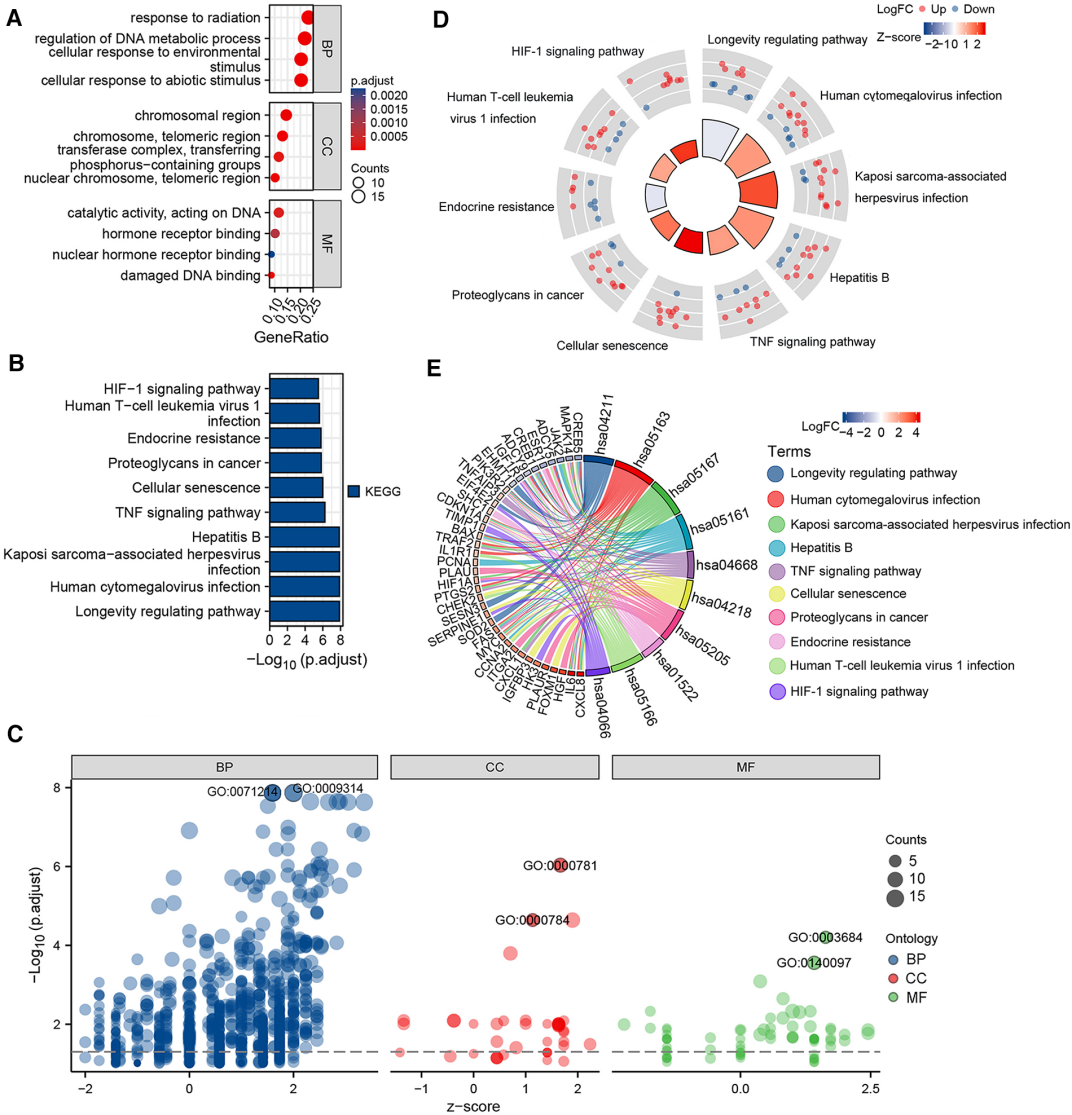
Gene ontology (GO) and Kyoto encyclopedia of genes and genomes (KEGG) enrichment analysis of 70 aging-related differentially expressed genes (DEGs). (**A**) Bubble plot of the GO enrichment analysis of aging-related DEGs. (**B**) Histogram of the KEGG pathway enrichment analysis of aging-related DEGs. (**C**) Bubble plot of the GO enrichment analysis of aging-related DEGs combined with logFC. (**D**) KEGG circle plot of aging-related DEGs combined with logFC. (**E**) KEGG chord diagram of aging-related DEGs combined with logFC.

The potential biological functions of the aging-related DEGs were analyzed by performing GO, KEGG, and GSEA enrichment analysis using R software. GO analysis showed that BPs of the aging-related DEGs were mainly focused on the response of cells to abiotic and environmental stimuli, as well as regulation of DNA metabolic processes; CCs involved the chromosomes, nuclear chromosomes, telomeric regions, transferase complexes, and transfer phosphorus-containing groups; and MF was focused on impaired DNA binding and hormone receptor binding (Figures 2A,C; Supplementary Table S1). KEGG pathway analysis showed that the aging-related DEGs were mainly enriched in the longevity regulating pathway, cellular senescence, tumor necrosis factor (TNF) signaling pathway, and hypoxia inducible factor-1 (HIF-1) signaling pathway (Figures 2B,D,E; Supplementary Table S2). GSEA of the upregulated genes in the GSE52093 dataset revealed that the aging-related genes, such as those involved in the p53 signaling pathway, were significantly enriched, while the enrichment of down-regulated genes was not significant (Supplementary Figure S2; Supplementary Table S3).

### 3.2. Correlation analysis and PPI network of aging-related DEGs

**Figure 3:**
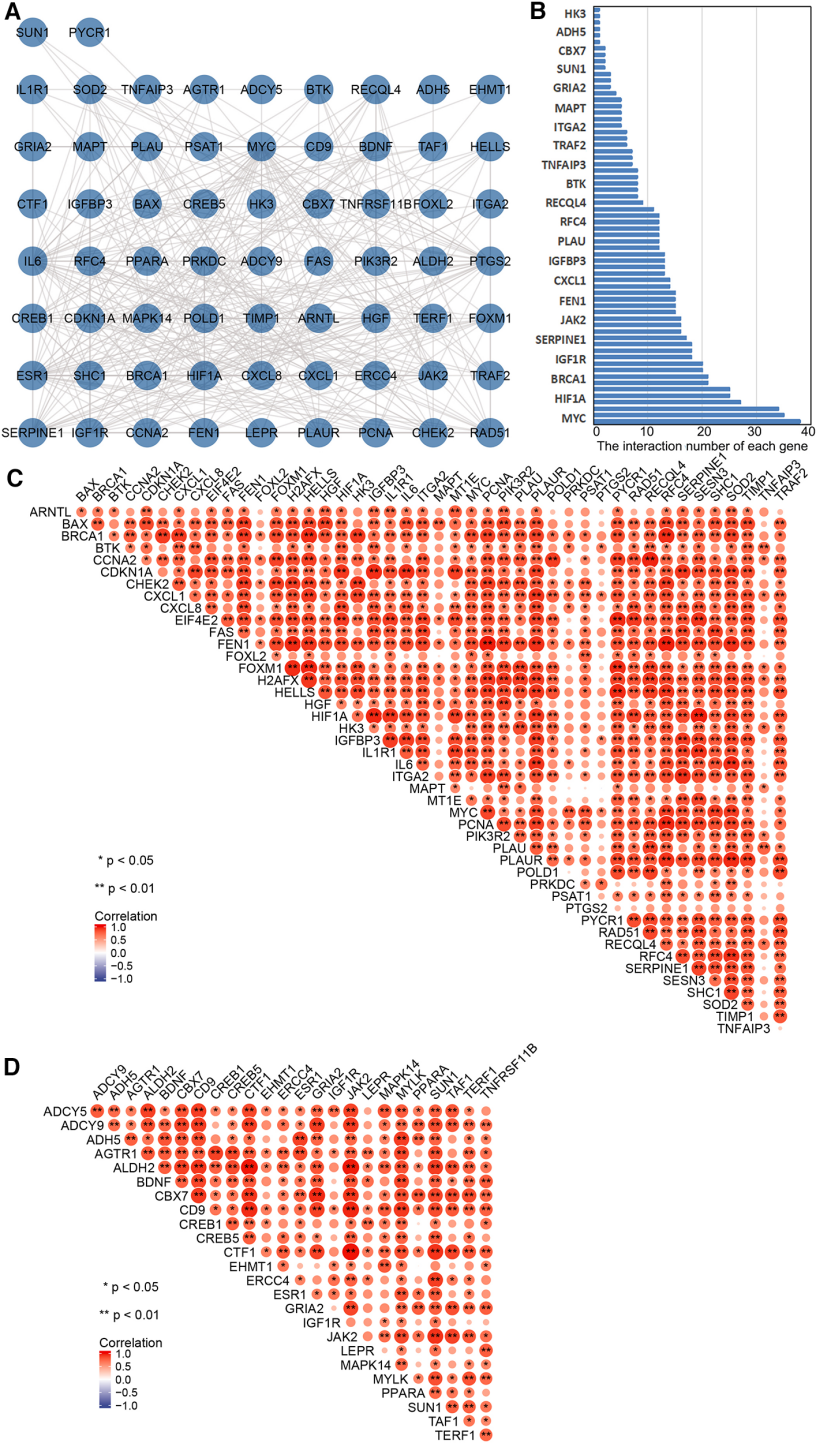
Correlation and protein–protein interaction (PPI) network analysis of aging-related differentially expressed genes (DEGs). (**A**) PPI network map of 70 aging-related DEGs in TAAD. (**B**) The interaction number of each aging-related DEGs. (**C**) Spearman correlation analysis of the 45 upregulated aging-related DEGs in TAAD. (**D**) Spearman correlation analysis of the 25 downregulated aging-related DEGs in TAAD.

PPI network analysis indicated an interaction of senescence-related genes (Figure 3A); the number of interactions for each gene is shown in Figure 3B. Correlation analysis showed relationships between 45 upregulated and 25 downregulated aging-related DEGs (Figures 3C,D). Overlapping genes were screened with five sequencing methods of the cytoHubba plugin in Cytoscape software; the top five overlapping genes—*MYC*, *IL6*, *HIF1A*, *ESR1*, and *PTGS2*—were taken as hub genes (Table 2).

**Table 2:** Differentially expressed aging-related genes screened by five methods of cytoHubba in the Cytoscape software package.

Degree	Closeness	EPC	MNC	Radiality
*MYC*	*MYC*	*MYC*	*MYC*	*MYC*
*IL6*	*IL6*	*ESR1*	*IL6*	*IL6*
*ESR1*	*ESR1*	*HIF1A*	*ESR1*	*ESR1*
*HIF1A*	*HIF1A*	*IL6*	*HIF1A*	*HIF1A*
*PTGS2*	*PTGS2*	*PTGS2*	*PTGS2*	*PTGS2*
*CXCL8*	*CXCL8*	*CDKN1A*	*CXCL8*	*CDKN1A*
*BRCA1*	*CDKN1A*	*CXCL8*	*BRCA1*	*CXCL8*
*CDKN1A*	*BRCA1*	*IGF1R*	*CDKN1A*	*IGF1R*
*IGF1R*	*IGF1R*	*BRCA1*	*IGF1R*	*MAPK14*
*CREB1*	*CREB1*	*MAPK14*	*CCNA2*	*CREB1*

### 3.3. Hubgenes and their interactions

**Figure 4:**
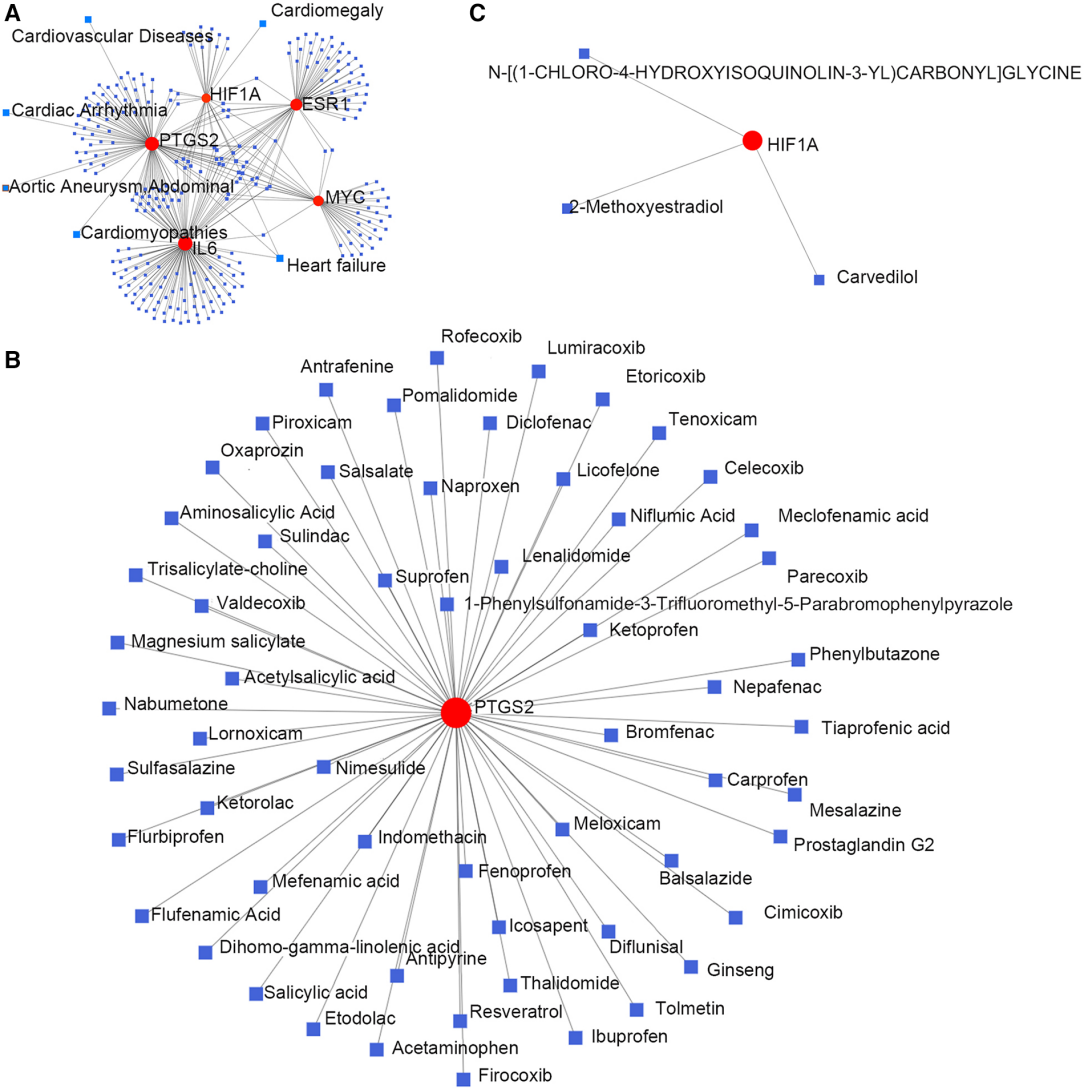
Hub genes and their interactions. (**A**) Hub genes–disease association information. (**B,C**) Hub genes–drug interactions.

Hub gene–disease association information and hub gene–drug interactions were constructed using the GSE52093 dataset. Gene–disease association information were as follows (Figure 4A): *HIF1A* is associated with heart failure and cardiac hypertrophy; *IL6* is associated with heart failure and cardiomyopathy; *PTGS2* is associated with aortic aneurysms, heart failure, cardiac arrhythmias, cardiomyopathy, and CVD. Gene–drug interactions were as follows: *PTGS2* may have interactions with 59 drugs, including aminosalicylic acid, acetaminophen, nabumetone, ketorolac, and tenoxicam (Figure 4B); *HIF1A* may have interactions with carvedilol, 2-methoxyestradiol, and N-[(1-chloro-4-hydroxyisoquinolin-3-YL) carbonyl] glycine (Figure 4C). This may provide new targets for the drug treatment of TAAD.

### 3.4. Verification of hubgene distribution and expression in the aging rat aorta by single-cell RNA sequencing

**Figure 5:**
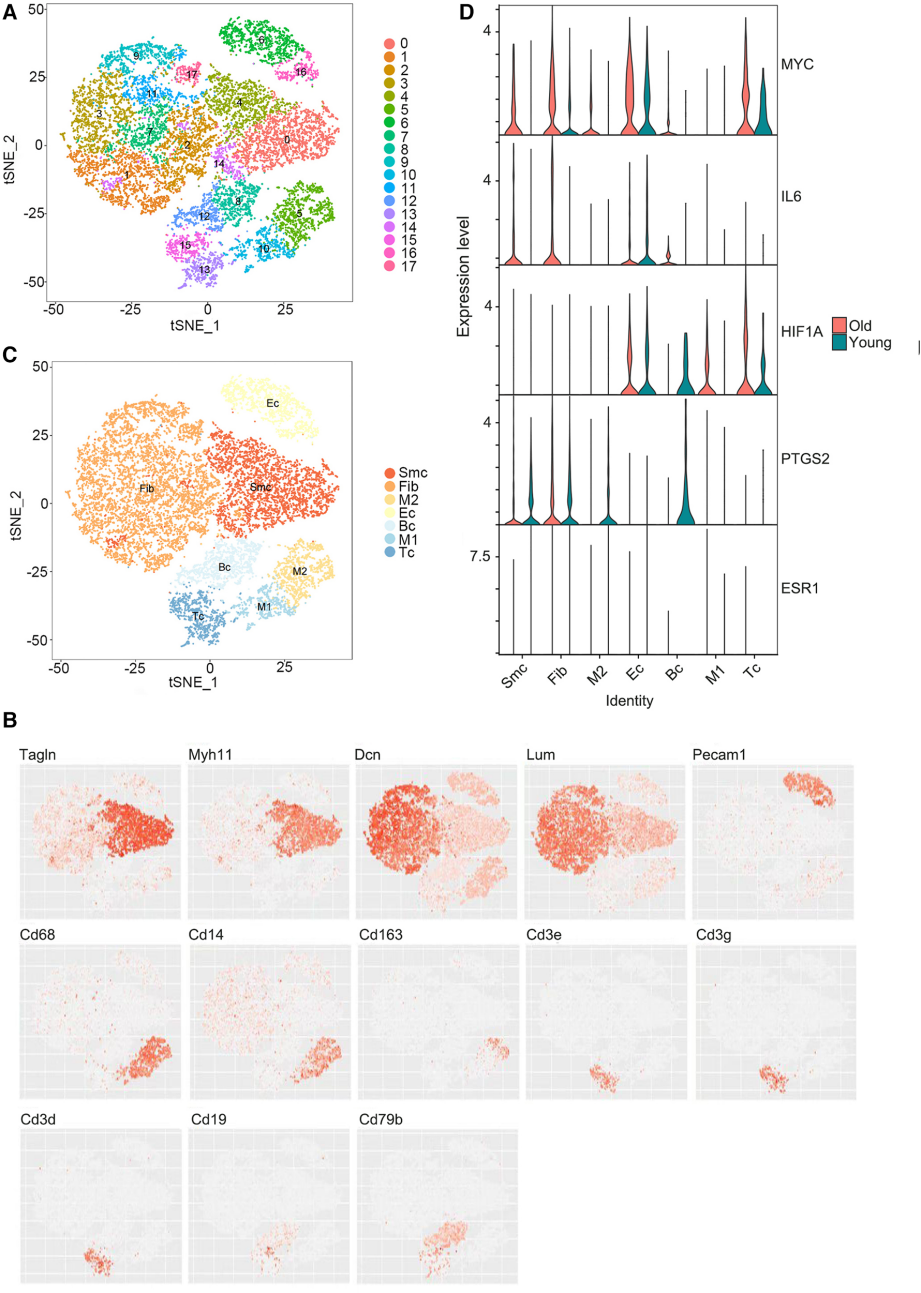
Single-cell RNA sequencing results. (**A**) Reduced dimensional plot of aortic tissue cell clustering t-SNE. (**B**) Reduced-dimensional plot of t-SNE for relative expression of cellular marker genes in different cell types. (**C**) Downscaled plots of t-SNE for aortic tissue cell subgroups. (**D**) Violin plots of hub genes expression in different cell types of aortic tissues.

Using the single-cell sequencing dataset GSE137869, four aortic samples (F-O, F-Y, M-O, and M-Y) were selected for single-cell sequencing analysis. The cells in the aortic tissue were clustered in descending order, which yielded 18 cell clusters (Figure 5A). t-SNE descending (Figure 5B) and violin plots (Supplementary Figure S3) were generated based on the expression of known cellular markers in different morphological cells. The cell clusters were divided into seven cell groups (Figure 5C): SMC (Tagln, MYH11), fibroblasts (Fib) (Dcn, Lum), EC (Pecam1), B cells (BC) (CD19, CD79b), T cells (TC) (CD3e, CD3g, CD3d), M1-type macrophages (CD68, CD14, CD163), and M2-type macrophages (CD68, CD14, CD163). The distribution and expression of the hub genes were compared in the aorta of young and old rats (Figure 5D; Supplementary Tables S4, S5), and this showed that *MYC* expression was upregulated in SMC, Fib, EC, and TC (*P* < 0.001) and downregulated in M2-type macrophages (*P* < 0.01) in the aorta of aging rats; *IL6* expression was upregulated in SMC, Fib (*P* < 0.001), and EC (*P* < 0.01) and downregulated in M1-type macrophages (*P* < 0.05); *HIF1A* expression was upregulated in M1-type macrophages (*P* < 0.05) and TC (*P* < 0.001), and downregulated in Fib (*P* < 0.001) and BC (*P* < 0.01); *PTGS2* expression was up-regulated in Fib (*P* < 0.001) and downregulated in SMC, BC, M2 macrophages (*P *< 0.001), EC, TC (*P *< 0.01), and M1-type macrophages (*P* < 0.05); *ESR1* expression was downregulated in Fib (*P* < 0.001).

### 3.5. Validation of hubgene expression in aging

**Figure 6:**
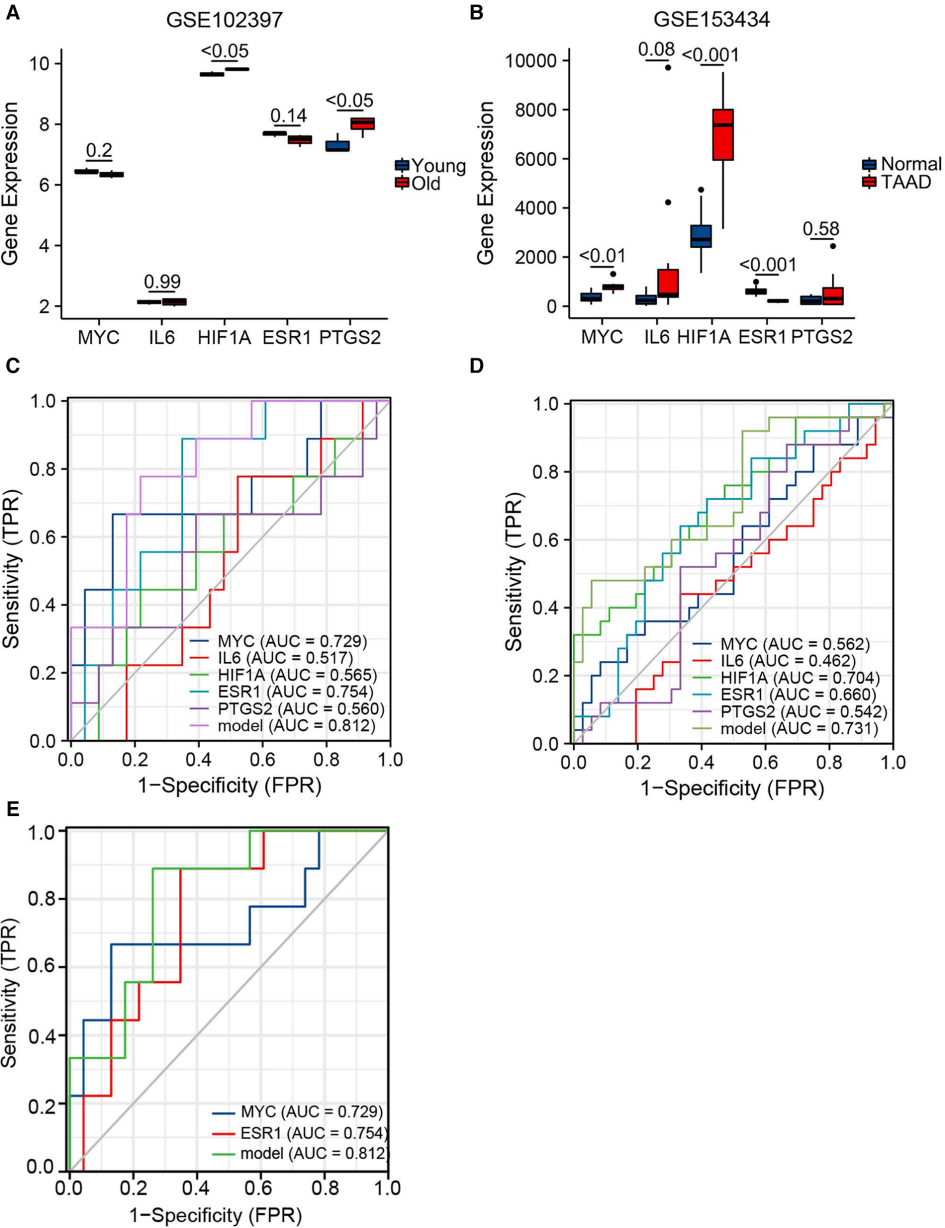
Validation of hubgene expression levels in aging and TAAD. (**A**) Grouped comparison plots of hub genes expression validation in the aging-related dataset GSE102397. (**B**) Grouped comparison plots of hub genes expression validation in the TAAD-related dataset GSE153434. (**C**) Diagnostic receiver operating characteristic (ROC) curves showing the independent and combined metrics for hub genes in the testing set. (**D**) Diagnostic ROC curves showing the independent and combined metrics in the training set. (**E**) Diagnostic ROC curves showing the independent and combined metrics for hub genes with AUC >0.7 in the testing set.

Expression levels of the top five hubgenes screened by Cytoscape software were verified in the aging-related datasets GSE102397 and the results were visualized using “ggplot2”. *HIF1A* and *PTGS2* expression was upregulated (*P* < 0.05) in GSE102397 (Figure 6A).

### 3.6. Validation of hubgene expression in TAAD

To verify the expression level of the hubgenes in TAAD-related datasets, the validation results were visualized using “ggplot2”. In GSE153434 (Figure 6B), the expression of *MYC* (*P *< 0.01) and *HIF1A* (*P *< 0.001) was upregulated, and *ESR1* (*P *< 0.001) was downregulated in the case group. The expression levels of hub genes were also examined in the coronary heart disease-related dataset GSE71226, the heart failure-related dataset GSE9128, and the aging cardiac tissue-related dataset GSE12480, and the results are shown in Supplementary Figure S4.

### 3.7. Diagnostic prediction of the hubgenes

Hub gene expression data in the GSE9106 dataset (testing set and training set) were selected and the “pROC” and “ggplot2” packages were used to draw a diagnostic ROC curve (Figures 6C,D). The area under the diagnostic ROC curve (AUC) showed that in the testing set the combined AUC of these five hubgenes was 0.812, while in the training set the combined AUC value was 0.731. This suggested that the combination of these five genes has a strong diagnostic power in the diagnosis of TAAD. A combined ROC with *MYC* and *ESR1* that had individual AUC > 0.7 was then performed and the results were equal to the AUC value of the combined ROC of all five hub genes (Figure 6E). Thus, these two genes may play a more important role in the diagnosis of TAAD.

## 4. Discussion

Aging is a major risk factor for TAAD (2, 3), and therefore exploring the relationship between aging and TAAD and investigate the mechanisms involved in the process are crucial for improving the diagnosis and treatment of TAAD. In this study, aging-related DEGs were identified and the HIF-1 signaling pathway was predicted to be a potential pathway that may be instrumental in aging-related TAAD. The study also revealed that the aging-related genes *MYC*, *IL6*, *HIF1A*, *ESR1* and *PTGS2* could be potential as a biomarker of aging-related TAAD.

The biological functions of aging-related DEGs in TAAD were explored through GO, KEGG, and GSEA, which revealed that these genes were predominantly enriched in pathways such as the HIF-1 signaling pathway, the p53 signaling pathway, and the TNF signaling pathway. HIF is a transcription factor that can respond to changes in effective oxygen in the cellular environment, especially to a reduction in oxygen or hypoxia. Previous research showed that HIF-1 signaling pathway is involved in the formation of arterial dissection (16) that HIF-1 signaling plays a key role in atheroma and atherosclerosis (17). Increased expression of HIF-1α has been found in human and experimental aneurysms, along with histological evidence of mural leukocyte infiltration and reduced angiogenesis (18). *HIF1A* participates in the HIF-1 signaling pathway and the p53 signaling pathway and induces p53 up regulation through HIF-1a under hypoxia (19). p53 induces cellular senescence and plays a pathological role in aging and age-related diseases such as heart failure (HF) and atherosclerosis (20, 21). In addition, the binding of TNF, also known as TNF-α, to the receptors activates the NF-kB and MAPK signaling pathways, which leads to the production of inflammatory cytokines and the secretion of matrix metalloproteinases (MMPs) and other extracellular matrix (ECM) degradation proteins, and accelerates TAAD formation and progression (22, 23). These reports help strengthen the inference that aging may aggravate TAAD by regulating the HIF-1 signaling pathway, etc.

*MYC*, *IL6*, *HIF1A*, *ESR1* and *PTGS2* were identified as hub genes through PPI analysis. *HIF1A* and *PTGS2* were validated in the aging dataset GSE102397 and *MYC*, *HIF1A* and *ESR1* were validated in the TAAD dataset GSE153434. *MYC* activation has been shown to leads to DNA damage, induction of p53, and massive apoptosis, with p53 activation mediating cellular senescence (24). In addition, MYC signaling participated in vascular smooth muscle cell (VSMC) dysfunction, vasoconstriction, and vascular remodeling in aortic dissection, and was increased in TAAD (25). In this study, single-cell sequencing of the aging rat aorta showed that MYC was expressed in the SMC, Fib, EC, etc. In the TAAD validation dataset GSE153434, MYC expression was significantly increased in TAAD. Therefore, we hypothesize that the increased expression of MYC not only induces cell senescence, but also contributes to molecular mechanisms of VSMC vasoconstriction and remodeling, thereby promoting TAAD.

It was previously reported that aging leads to elevated levels of the inflammatory cytokine *IL6* in the aorta (26), which consistent with our aging rat aortic single cell sequencing results that *IL6* expression was up-regulated in SMC, Fib and EC. In addition, our results indicate that the expression of *IL6* is increased in TAAD. A previous study demonstrated that *IL6* could regulate autophagy by enhancing 4B cysteine peptidase (ATG4B), decreasing the expression of the VSMC contractile proteins α-SMA and SM22α, and promoting TAAD (27). We suggest that aging leads to elevated IL6 expression level in aortic SMCs, which promotes TAAD by inducing the degradation of the VSMC.

HIF1A is the alpha subunit of HIF-1, which is involved in energy metabolism, angiogenesis, and apoptosis through the activation of gene transcription. Studies in elderly rhesus monkeys have shown a positive association between HIF1A and aging (28), which is comparable to gene expression data in our study. Single-cell sequencing of the aging rat aorta showed that HIF1A expression was upregulated in M1-type macrophages and TC, and downregulated in Fib and BC, which revealed elevated expression of HIF1A in inflammatory cells in the aging aorta. Previous studies showed that *HIF1A* expression was positively correlated with the inflammatory degree of the disease (18, 29). HIF-1α activation also promotes the progression of aortic dissection through vascular inflammation, extracellular matrix degradation, and rupture of the elastic fibers (30). In the validation TAAD dataset GSE153434, HIF1A expression was significantly upregulated in patients with TAAD compared with the control group. In addition, the diagnostic ROC curve analysis results of *HIF1A* in the training set was 0.704, which had diagnostic significance. Thus, we hypothesize that aging may induce HIF1A overexpression in inflammatory cells and activate the HIF-1 pathway, thereby regulating the inflammatory response to promote TAAD.

In our study, single-cell sequencing of the aging rat aorta showed that *PTGS2* expression was predominantly upregulated in Fib and downregulated in SMC, etc., and *PTGS2* expression was upregulated in aging aortas compared with the young aortas. Previous studies have shown that PTGS2 is one of the risk factors of CVD (31). *PTGS2* was downregulated in the late stage of atherosclerosis, and the expression of PTGS2 was positively correlated with the severity of atherosclerosis (32). Unlike other hub genes, single-cell sequencing of ESR1 in the aging rat aorta showed that ESR1 expression was down-regulated in all cell types. ESR1 is estrogen receptor 1, also known as ERα, and is expressed in human and animal vascular ECs, VSMCs, and cardiomyocytes (33). In atherosclerosis, ERα inhibits VSMC differentiation and lipid aggregation and enhances antioxidant activity (34). Upregulation of ERα was also observed to inhibit high glucose-induced VSMC proliferation by inhibiting ROS-mediated ERK activation (35). The diagnostic ROC curve analysis results of *ESR1* in the testing set in this study was 0.754, which had diagnostic significance. Therefore, we suggest that aging leads to downregulation of ESR1 expression in cells such as SMC and EC, which fails to inhibit cellular dysfunction, VSMC proliferation and differentiation, and antioxidant capacity, thereby promoting TAAD.

To explore whether the hub genes had specific diagnostic ability for age-related TAAD, the expression levels of the hub genes were also validated in other datasets related to age-related CVDs such as coronary heart disease, heart failure and aging cardiac tissue. There was no significant difference in the expression of the hub genes in coronary heart disease, but *MYC* and *PTGS2* expression was significantly increased in cardiac tissue and non-ischemic heart failure. This suggests that *MYC* and *PTGS2* may have some significance in non-ischemic heart failure. However, unlike heart failure, *MYC*, *ESR1* and HIF-1 pathway have main functions in TAAD.

There were some limitations to the this study. The results were only obtained by bioinformatic analysis and were not confirmed by molecular experiments. Aortas from aging and young patients with TAAD are difficult to obtain, and aging animal models of TAAD are also challenging to perform, however, this is an important direction for our future work.

## 5. Conclusion

This study reports that the HIF-1 signaling pathway may play a significant role in TAAD and aging, and may serve as a key pathway for an in-depth study of the disease mechanisms. *MYC* and *ESR1* have certain diagnostic value for age-related TAAD and have potential as aging-related TAAD biomarkers. Extensive *in vivo* and *ex vivo* clinical studies as well as experimental validation are required in the future to confirm the expression and mechanism of action of these genes in TAAD.

## Data Availability

The datasets presented in this study can be found in online repositories. The names of the repository can be found in the article.
