# Supplementary material for: Bioinformatics analysis of aging-related genes in thoracic aortic aneurysm and dissection
Source: Front Cardiovasc Med. 2023 May 22;10:1089312. doi: 10.3389/fcvm.2023.1089312 (PMC10239936; doi:10.3389/fcvm.2023.1089312)
Supplement: Supplementary file 1 [file Table1.docx]

| Ontology | ID | Description | GeneRatio | BgRatio | pvalue | p.adjust | qvalue |
| --- | --- | --- | --- | --- | --- | --- | --- |
| BP | GO:0009314 | response to radiation | 16/69 | 448/18670 | 5.52e-12 | 1.37e-08 | 8.03e-09 |
| BP | GO:0071214 | cellular response to abiotic stimulus | 14/69 | 331/18670 | 1.48e-11 | 1.37e-08 | 8.03e-09 |
| BP | GO:0104004 | cellular response to environmental stimulus | 14/69 | 331/18670 | 1.48e-11 | 1.37e-08 | 8.03e-09 |
| BP | GO:0051052 | regulation of DNA metabolic process | 15/69 | 429/18670 | 3.76e-11 | 2.26e-08 | 1.32e-08 |
| CC | GO:0000781 | chromosome, telomeric region | 9/69 | 161/19717 | 4.81e-09 | 9.23e-07 | 6.53e-07 |
| CC | GO:0000784 | nuclear chromosome, telomeric region | 7/69 | 125/19717 | 2.71e-07 | 2.29e-05 | 1.62e-05 |
| CC | GO:0098687 | chromosomal region | 10/69 | 349/19717 | 3.57e-07 | 2.29e-05 | 1.62e-05 |
| CC | GO:0061695 | transferase complex, transferring phosphorus-containing groups | 8/69 | 259/19717 | 3.33e-06 | 1.60e-04 | 1.13e-04 |
| MF | GO:0003684 | damaged DNA binding | 6/69 | 65/17697 | 1.94e-07 | 6.26e-05 | 4.17e-05 |
| MF | GO:0140097 | catalytic activity, acting on DNA | 8/69 | 213/17697 | 1.72e-06 | 2.78e-04 | 1.85e-04 |
| MF | GO:0051427 | hormone receptor binding | 7/69 | 185/17697 | 7.60e-06 | 8.19e-04 | 5.44e-04 |
| MF | GO:0035257 | nuclear hormone receptor binding | 6/69 | 152/17697 | 2.79e-05 | 0.002 | 0.001 |
