# Supplementary material for: Bioinformatics analysis of aging-related genes in thoracic aortic aneurysm and dissection
Source: Front Cardiovasc Med. 2023 May 22;10:1089312. doi: 10.3389/fcvm.2023.1089312 (PMC10239936; doi:10.3389/fcvm.2023.1089312)
Supplement: Supplementary file 2 [file Table2.docx]

| Ontology | ID | Description | GeneRatio | BgRatio | pvalue | p.adjust | qvalue |
| --- | --- | --- | --- | --- | --- | --- | --- |
| KEGG | hsa04211 | Longevity regulating pathway | 11/63 | 89/8076 | 5.96e-11 | 1.31e-08 | 5.46e-09 |
| KEGG | hsa05163 | Human cytomegalovirus infection | 15/63 | 225/8076 | 1.11e-10 | 1.31e-08 | 5.46e-09 |
| KEGG | hsa05167 | Kaposi sarcoma-associated herpesvirus infection | 14/63 | 193/8076 | 1.66e-10 | 1.31e-08 | 5.46e-09 |
| KEGG | hsa05161 | Hepatitis B | 13/63 | 162/8076 | 2.31e-10 | 1.37e-08 | 5.71e-09 |
| KEGG | hsa04668 | TNF signaling pathway | 10/63 | 112/8076 | 1.21e-08 | 5.74e-07 | 2.40e-07 |
| KEGG | hsa04218 | Cellular senescence | 11/63 | 156/8076 | 2.55e-08 | 1.01e-06 | 4.20e-07 |
| KEGG | hsa05205 | Proteoglycans in cancer | 12/63 | 205/8076 | 4.44e-08 | 1.50e-06 | 6.28e-07 |
| KEGG | hsa01522 | Endocrine resistance | 9/63 | 98/8076 | 5.42e-08 | 1.61e-06 | 6.70e-07 |
| KEGG | hsa05166 | Human T-cell leukemia virus 1 infection | 12/63 | 219/8076 | 9.22e-08 | 2.43e-06 | 1.01e-06 |
| KEGG | hsa04066 | HIF-1 signaling pathway | 9/63 | 109/8076 | 1.37e-07 | 3.16e-06 | 1.32e-06 |
