# Supplementary material for: Bioinformatics analysis of aging-related genes in thoracic aortic aneurysm and dissection
Source: Front Cardiovasc Med. 2023 May 22;10:1089312. doi: 10.3389/fcvm.2023.1089312 (PMC10239936; doi:10.3389/fcvm.2023.1089312)
Supplement: Supplementary file 3 [file Table3.docx]

**GSEA enrichment analysis of up-regulated genes**

| ID | ES | NES | P.adjust | FDR |
| --- | --- | --- | --- | --- |
| KEGG_CELL_CYCLE | 0.637 | 1.677 | 0.025 | 0.022 |
| KEGG_CYTOKINE_CYTOKINE_RECEPTOR_INTERACTION | 0.598 | 1.580 | 0.025 | 0.022 |
| KEGG_ECM_RECEPTOR_INTERACTION | 0.773 | 1.854 | 0.025 | 0.022 |
| KEGG_FOCAL_ADHESION | 0.519 | 1.377 | 0.025 | 0.022 |
| KEGG_P53_SIGNALING_PATHWAY | 0.689 | 1.733 | 0.025 | 0.022 |

**GSEA enrichment analysis of down-regulated genes**

| ID | ES | NES | P.adjust | FDR |
| --- | --- | --- | --- | --- |
| KEGG_VASCULAR_SMOOTH_MUSCLE_CONTRACTION | -0.664 | -1.597 | 0.068 | 0.065 |
| NABA_CORE_MATRISOME | -0.672 | -1.660 | 0.068 | 0.065 |
| NABA_ECM_GLYCOPROTEINS | -0.645 | -1.571 | 0.068 | 0.065 |
| NABA_MATRISOME | -0.621 | -1.583 | 0.068 | 0.065 |
| NABA_MATRISOME_ASSOCIATED | -0.582 | -1.464 | 0.068 | 0.065 |
