# Supplementary material for: Bioinformatics analysis of aging-related genes in thoracic aortic aneurysm and dissection
Source: Front Cardiovasc Med. 2023 May 22;10:1089312. doi: 10.3389/fcvm.2023.1089312 (PMC10239936; doi:10.3389/fcvm.2023.1089312)
Supplement: Supplementary file 6 [file Table6.docx]

Supplementary Material

# Supplementary Figures and Tables

## Supplementary Figures


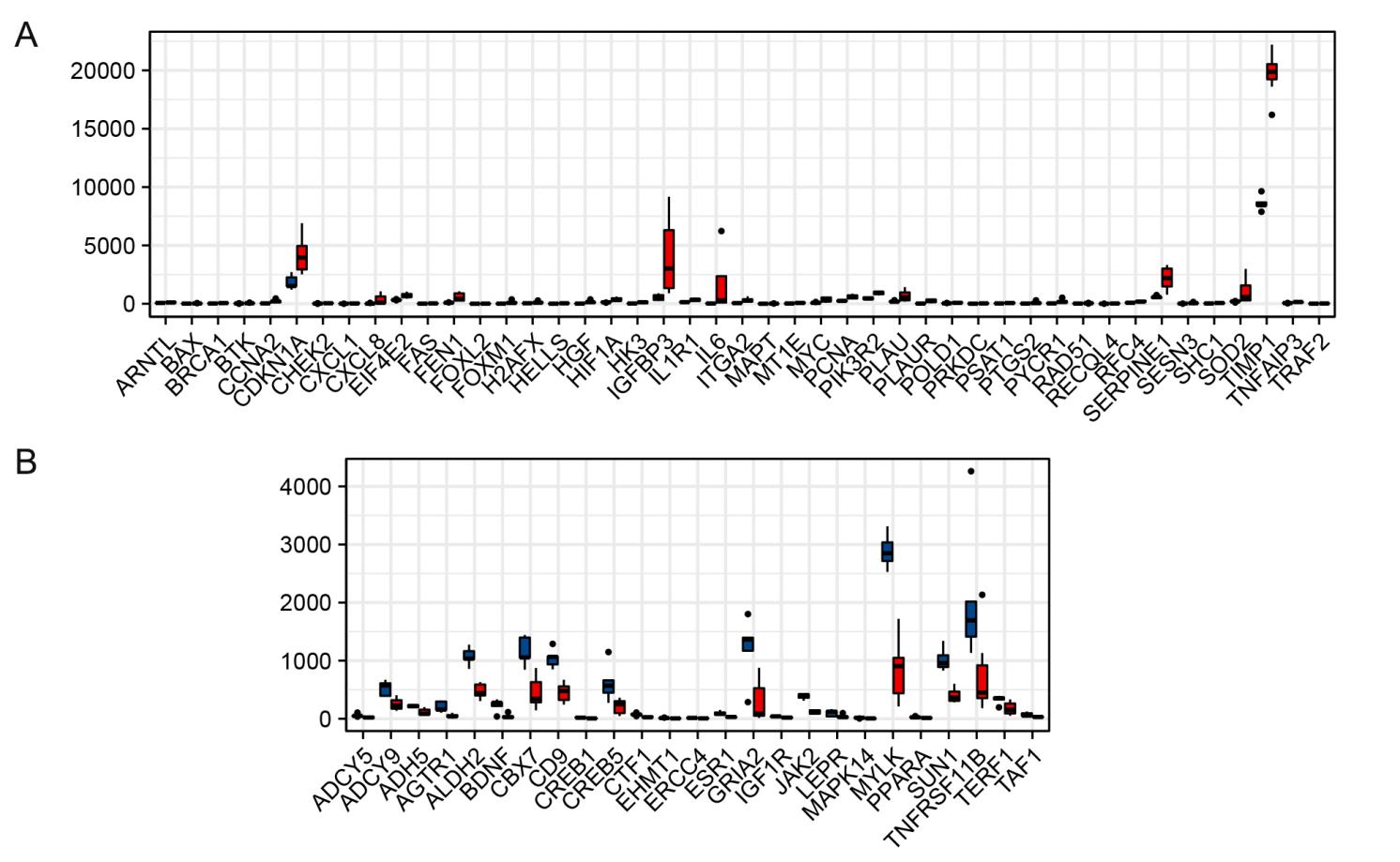


**Supplementary Figure 1.** Boxplots of expression of 70 aging-related genes in TAAD. **(A)**Boxplots of expression of up-related genes.**(B)**Boxplots of expression of down-related genes.


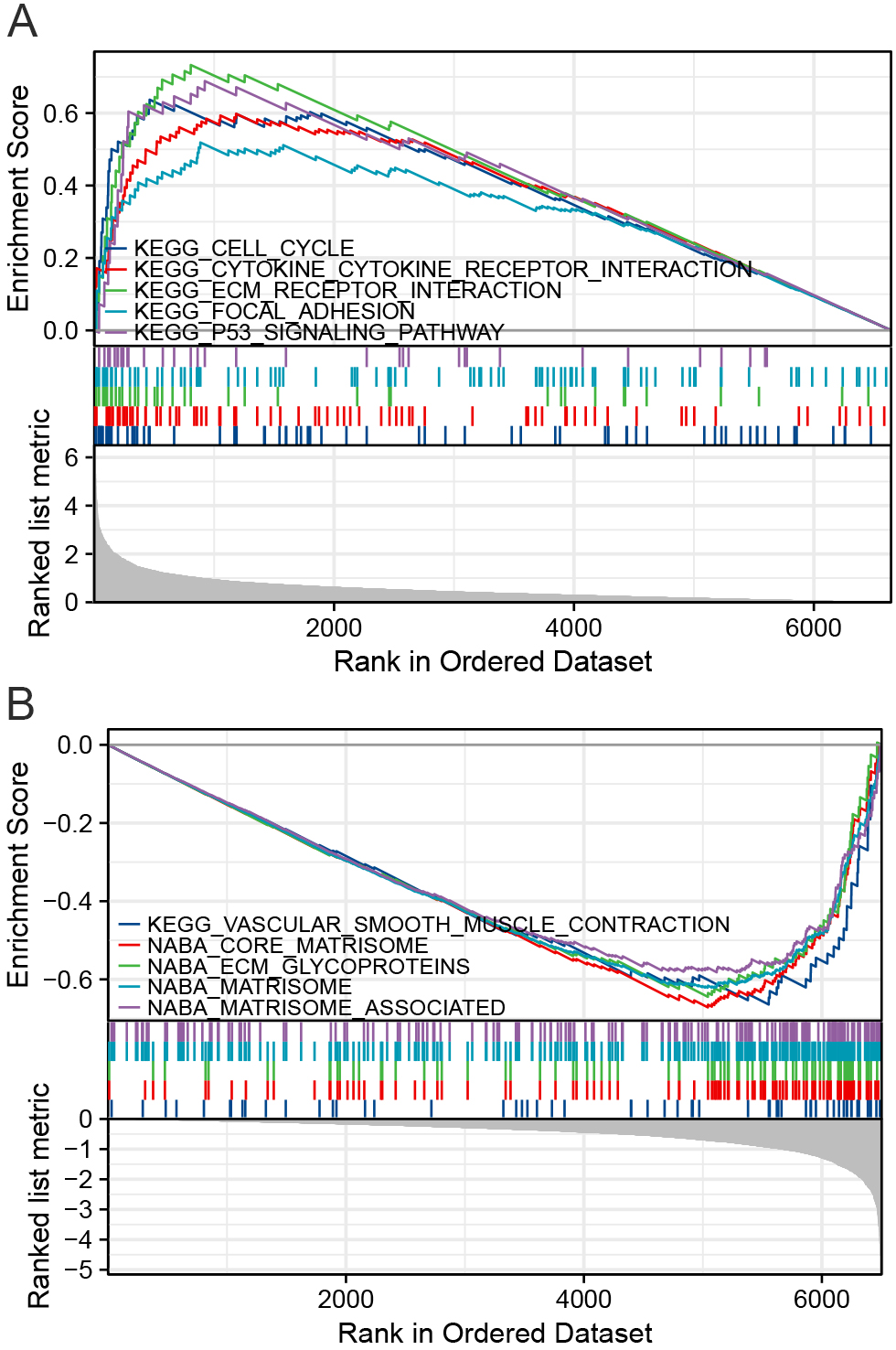


**Supplementary Figure 2.**GSEA enrichment analysis of GSE52093 gene. **(A)** GSEA enrichment analysis of GSE52093 up-regulated genes. **(B)** GSEA enrichment analysis of GSE52093 down-regulated genes.

**
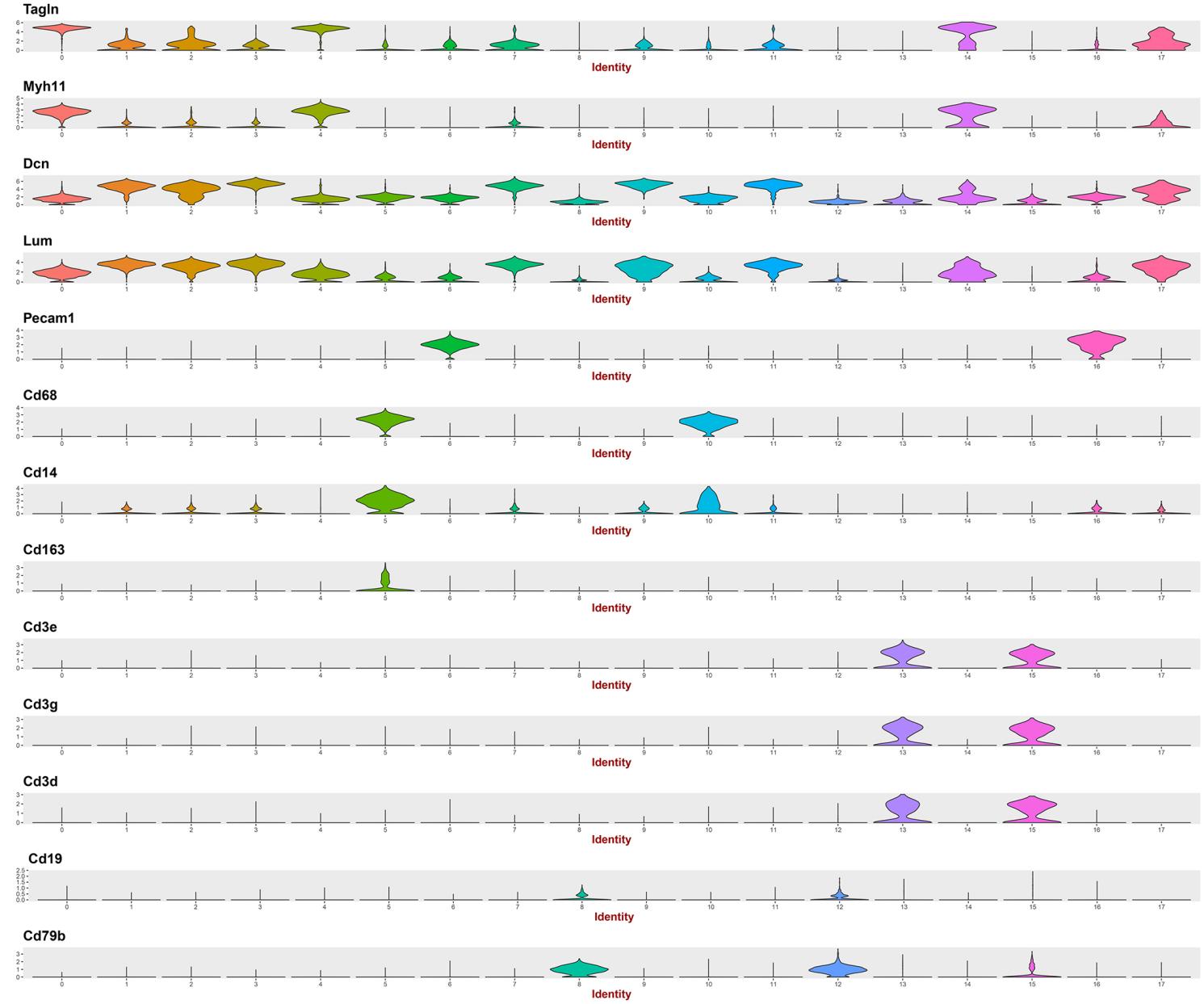
**

**Supplementary Figure 3.**Violin plots of the expression levels of known cellular markers in aortic tissue cells in different cell types.


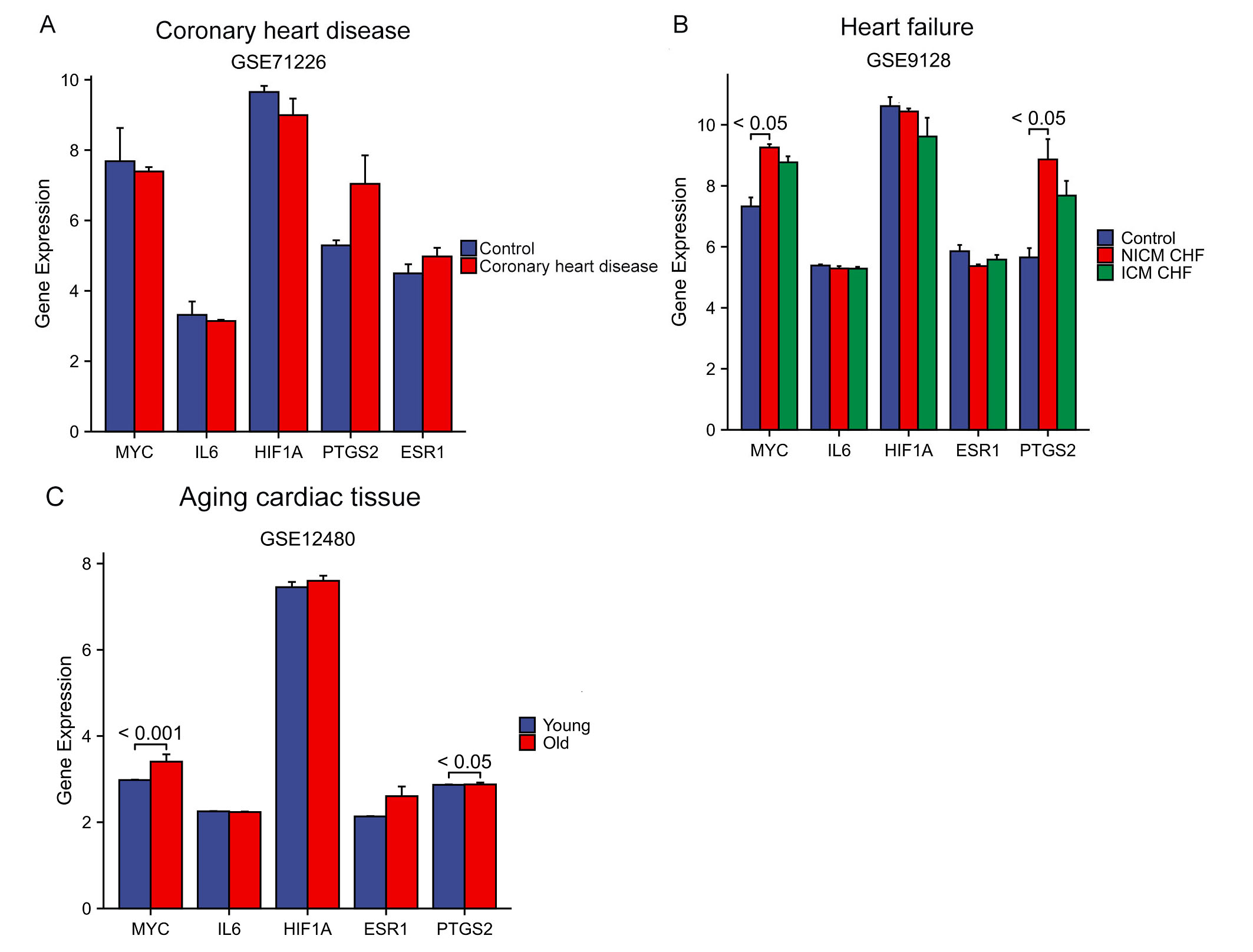
**Supplementary Figure 4. (A)** Grouped comparison plots of hubgene expression validation in coronary heart disease-related dataset GSE71226. **(B)** Grouped comparison plots of hubgene expression validation in heart failure-related dataset GSE9128. **(C)** Grouped comparison plots of hubgene expression validation in aging cardiac tissue.
